# “It’s a hard thing to manage when you’re homeless”: the impact of the social environment on smoking cessation for smokers experiencing homelessness

**DOI:** 10.1186/s12889-019-6987-7

**Published:** 2019-05-24

**Authors:** Rebekah Pratt, Claire Pernat, Linda Kerandi, Azul Kmiecik, Cathy Strobel-Ayres, Anne Joseph, Susan A. Everson Rose, Xianghua Luo, Ned Cooney, Janet Thomas, Kola Okuyemi

**Affiliations:** 1 Program in Health Disparities Research, Department of Family Medicine and Community Health, University of Minnesota, 717 Delaware Street, Minneapolis, MN 55414 USA; 2 Department of Medicine, University of Minnesota, 401 East River Parkway, Minneapolis, MN 55455 USA; 3 Department of Medicine & Program in Health Disparities Research, University of Minnesota, Minneapolis, USA; 4 Division of Biostatistics, School of Public Health and Masonic Cancer Center, University of Minnesota, 420 Delaware Street SE, MMC 303, Minneapolis, MN 55455 USA; 5 Department of Psychiatry, Yale University School of Medicine, 300 George Street #901, New Haven, CT 06511 USA; 6 Department of Family & Preventive Medicine, University of Utah, 375 Chipeta, Suite A, Salt Lake City, UT 84108 USA

**Keywords:** Smoking cessation, Homelessness, Randomized control trial, Qualitative, Participant experience, Social environment

## Abstract

**Background:**

Up to 80% of the adult homeless population use tobacco, and smoking cessation programs could offer an important opportunity to address preventable mortality and morbidity for this population. This population faces serious challenges to smoking cessation, including the impact of the social environment.

**Methods:**

Forty participants (11 female; 29 male) from an ongoing smoking cessation randomized clinical trial conducted at 2 urban homeless shelters in the Upper Midwest were invited to take part in semi-structured interviews in 2016–2017. An interviewer used a semi-structured interview guide asking participants to describe their experience of how the social environment impacted their attempt to quit smoking.

**Results:**

Participants described feeling pressure to smoke and drink in and around shelters, and that this pressure had led some to start smoking or resume smoking, along with making it very challenging to quit. Participants described being motivated to quit, and seeing smoking cessation as positively impacting the time and focus they felt they had for finding housing. However many felt more interested in reducing their smoking, rather than quitting.

**Conclusions:**

Addressing smoking cessation for people experiencing homelessness is both an important public health opportunity, and a challenge. There is a need to consider cessation in the context of the social and environmental factors impacting smokers who are experiencing homelessness. In particular, there is a need to address the collective value placed on smoking in social interactions. Despite these challenges, there are high levels of motivation and interest in addressing smoking.

**Trial registration:**

NCT01932996. Date of registration 30th August 2013. Prospectively registered.

## Background

Cigarette smoking among people experiencing homelessness is both a serious problem and an important public health opportunity. Approximately 4.2% of adults residing in the United States will experience homelessness within their lifetime, and 1.5% within the last year [1]. Approximately 70 to 80% of the adult homeless population smokes cigarettes [2], compared to around 15% percent of the general population [3]. Smoking cessation programs could offer an important opportunity to address preventable mortality and morbidity for this population [4]. In this paper, we present qualitative findings from Power to Quit 2 (PTQ2), a smoking cessation randomized clinical trial for homeless smokers who also use alcohol. Our work has been influenced by social ecological approaches, which consider the role of individual health behavior, such as smoking cessation, in the context of interpersonal, community, cultural and structural barriers [5, 6].

Studies with smokers from low socioeconomic groups show that there can be feelings of shame and stigma around smoking [7]. However, when they are in environments that have pro-smoking norms, feelings of stigma and shame around smoking are reduced [8]. Another barrier to smoking cessation can be the presence of peers who smoke [9], particularly when smoking plays an important role in social interactions [10]. Shelter environments are often places where smoking is experienced as normative [10]. In one study, close shelter proximity was negatively associated with successful quit attempts, particularly immediately following the attempt to quit [11]. Additionally, there is a relationship between certain types of housing and the use of tobacco [2], with stable housing positively associated with abstinence outcomes [12]. These factors put the homeless population at a disadvantage in comparison to the general smoking population, when attempting to quit [13]. Additionally, some people experiencing homelessness describe smoking initiation while residing in a homeless shelter, where there might be limited access and care, unstructured schedules, fewer stress coping outlets, and a high social value on smoking [13].

The motivation to quit smoking has been routinely underestimated by professionals providing care for smokers experiencing homelessness due to assumptions that the barriers experienced in the social environment and the difficulties of homelessness would lead to little interest or motivation in smoking cessation [14–16]. However homeless smokers have reported being interested in cessation [10, 16]. Studies disagree on the extent of motivation to quit smoking [17], but even if motivation is similar to the general population, the challenges for homeless smokers differ drastically from the general population [2]. Research with people experiencing homelessness has reported that the stress of homelessness itself is seen as a barrier to cessation [9, 16]. There is also indication from smokers experiencing homelessness that reduction strategies might be preferred to cessation [18], due to the challenges of quitting while experiencing homelessness. Additionally, 95% of homeless smokers have a history of alcohol abuse or illicit drug use [19], and there may be value in considering smoking alongside other challenges that may influence the sense of agency to act successfully on the motivation to quit.

Addressing these barriers and tailoring interventions to fit the needs of homeless smokers, have been identified as important to increase self-efficacy for quitting [11, 18], although there remains some concern that stress from being homeless would be further exacerbated by attempts at quitting [20]. Despite these concerns about smoking cessation for smokers experiencing homelessness, attempts have been made to address this important public health issue. Nonetheless, smoking cessation intervention studies for people experiencing homelessness are limited, tend to be small, and have not substantially impacted quit rates [21, 22].

Power to Quit, one of the largest studies to date, tested the use of motivational interviewing alongside nicotine replacement therapy, which was not sufficient to significantly impact quit rates [13]. Those that did quit, however, also reduced alcohol consumption, suggesting that smoking and drinking may be best addressed concurrently [23]. PTQ2 builds on the work of Power to Quit, and tests the value of addressing alcohol use alongside smoking cessation for people experiencing homelessness [24, 25].

To better understand the experience of participating in a smoking cessation trial, 40 study participants took part in one-on-one interviews after their participation in the study intervention. The interviews explored individual views on participating in a smoking cessation intervention, perceived research priorities for issues impacting people experiencing homelessness, and their experiences of how the social and physical environment impacts participation in a smoking cessation intervention. In this paper, we present findings on participant experiences of their social and physical environment and their attempts to quit smoking. The study aim was to understand the important interplay between smoking cessation and the social environment for smokers experiencing homelessness. This will ultimately help with continuing to design cessation studies and programs that can meet the public health opportunity of providing smokers experiencing homelessness with tailored programs that are responsive to the challenges they face.

## Methods

### Parent study description

PTQ2 is a smoking cessation randomized clinical trial targeting smoking and alcohol use. The full design and methods are described elsewhere [25]. At the time these interviews were conducted, PTQ2 was utilizing a three arm study design, where participants were randomized to receive a) standard care in the form of brief counseling, or b) an intensive smoking cessation intervention focused cognitive behavioral therapy (CBT), coupled with motivational interviewing (MI), or c) an intensive smoking and alcohol cessation focused CBT/MI. The final outcome measure, biochemically verified cessation, was collected at 52 weeks. Due to challenges with recruitment, the study protocol was amended in 2017 to be a two-arm trial comparing standard care with a smoking and alcohol focused CBT intervention. Additionally, the final outcome measurements were moved to week 26. This group of participants was interviewed at and around their 26-week data collection point.

### Study population

Qualitative data were gathered using one-on-one semi-structured interviews from December 2016 to April 2017. A convenience sample of 40 participants was recruited to take part in the interviews. Interviews were conducted with both control arm and intervention arm participants. Fifteen standard care, 13 intensive smoking and 12 intensive smoking and alcohol arm participants were recruited.

### Study instrument

A semi-structured interview guide was developed by the research study team (see Table 1). The interview guide consisted of 17 questions on the topics of the experience of attempting to quit smoking during the study, their experience of the study intervention, and their views on important topics for future research. Interviews took between 20 to 60 min. For the purposes of this manuscript, a subset of data pertaining the impact of the social and environmental factors influencing smoking has been analyzed, along with data on potential future research. Data on the PTQ2 intervention implementation is presented elsewhere.

**Table 1:** Semi-structured interview guide

Semi-structured Interview Guide	
1. Can you describe to me what got you interested taking part in the PTQ II study?	
2. What were you hoping to get out of taking part in the study?	
3. What kinds of activities were you doing in the study?	
4. You mentioned you received (education/sessions on smoking/sessions on smoking and alcohol) as part of the study. What was your overall impression of doing these activities?	
5. How did you feel about the amount of education or counselling you received?	
6. Did you use the patch/gum/lozenge? If you didn’t use them every day for 12 weeks, what were some of the things that kept you from doing so?	
7. Were there any parts of the study activities you particularly liked?	
8. Were there any parts of the study activities you particularly did not like?	
9. How was it hard, or easy, to make it along to counselling sessions?	
10. How would you have felt about it if you had been offered an opportunity to do the counselling by phone?	
11. Did the sessions have any impact on your (smoking or smoking and drinking)?	
12. What could have made the sessions even better for you?	
13. In general, do you have any views on how dealing with homelessness impacts the ability of people to take part in studies like this?	
14. Did the counselling sessions have any impact on other aspects of your life, aside from the smoking/smoking and drinking?	
15. What did you think about the study questionnaires you were asked to complete?	
16. What did you think about the incentives you received?	
17. Finally what other topics do you think we should research to help us address smoking for people experiencing homelessness?	

### Data collection

At the time of consenting to the study, participants in PTQ2 were informed that they might be asked to participate in an interview. Potential participants were approached by research study staff when the participant was about to complete their week 26 of study participation. The participants were compensated with a $20 gift card for their time. To facilitate unbiased answers to questions about the study and the study team, the interviewer was a trained individual who was not part of the study intervention staff. The interviews were conducted in one of two urban shelters at which the study team was delivering the intervention.

### Data analysis

Interviews were recorded and then transcribed verbatim. The qualitative data were analyzed using NVivo 11 [26]. Three members of the research team (RP, PhD, AK, MPH, and CP, undergrad student) systematically coded the transcripts, and double coded a sub-set of data to ensure consistency. The coders met regularly to review emerging codes and come to a consensus on the emerging codebook. AK led the comprehensive initial coding process, with in-depth reviewing with RP. The emerging codes were shared with the study investigator team and study staff for feedback, allowing for further checking of interpretation of themes. RP and CP conducted a further analysis on the sub-set of the data that concerned the social environment. CP recoded the subset of data, creating a further and more detailed subset of themes, in collaboration with RP. The coding was guided by the social constructivist approach to grounded theory, where themes and sub-themes were developed from the data, while also considering the data in the broader context of the literature [27, 28]. In particular, the impact of social ecological approaches was central to considering the broader social and environmental context of the study participants. Discussions with a broader group of members of the research team on the emerging analysis further validated the rigor of the qualitative analysis. Data for this manuscript represents a subset of the full data set and codebook, as it related to social and environmental themes. The team chose to not prevent the frequency of themes as part of the results, but have noted general frequency, such as ‘many’ or ‘few’ to provide context for the reader. Data pertaining to the participant’s experiences of the study implementation is reported elsewhere.

### Human subjects

The University of Minnesota Institutional Review Board provided ethical approval for the conduct of this study.

## Results

### Demographics

Participant demographics were assessed at the time of study enrollment (see Table 2). Of the 40 participants who elected to take part in the interview, 11 were female, and 29 were male. Thirty-two participants identified as African American/Black, 6 as White, 1 as Native American/Alaska Native, and 1 as more than one race. At study enrollment, housing stability as assessed by self-report on a scale of 0 (not at all stable) to 10 (extremely stable); the mean (±SD) response was 3.53 ± 3.48 (range, 0 to 10). While there was a range of experiences, most participants were unemployed. Participants smoked an average of 14.6 ± 8.3 (range 2.5 to 40) cigarettes at their eligibility screening and just over half had their first cigarette of the day within 15 min of waking. Alcohol use severity was measured using the World Health Organization Alcohol Use Disorder Identification Test (AUDIT) [29, 30], a 10 question scale that measures drinking behavior, dependence, and consequences related to drinking. Participant scores averaged 14.9 ± 4.87 (range 7 to 24) which placed them in the risky/hazardous or high-risk/harmful alcohol use risk levels.

**Table 2:** Sample Demographics/Baseline Characteristics

	Mean ± SD (range) or *n* (%)
N	40
Study randomization arm	
A: Standard Care	15 (37.5%)
B: Intensive Smoking Intervention	13 (32.5%)
C: Intensive Smoking and Alcohol Intervention	12 (30.0%)
Age	50.20 ± 9.2 (29.6–69.5)
Sex	
Male	29 (72.5%)
Female	11 (27.5%)
Cigarettes smoked per day (on eligibility survey)^a^	14.6 ± 8.3 (2.5–40)
Housing situation (at eligibility survey)	
Emergency or overnight shelter	23 (57.5%)
Campsite, vehicle, abandoned building/house, parking garage, or on the street	7 (17.5%)
Transitional or supportive housing, long-term shelter	5 (12.5%)
Staying with relative, friend, or other people/double-up – less than 3 months at the same place	5 (12.5%)
Housing stability (self-rating from 0-not at all stable to 10-extremely stable)	3.53 ± 3.48 (0–10)
Race	
African American or Black	32 (80.0%)
Native American/Alaskan Native	1 (2.50%)
White	6 (15.0%)
More than 1 race	1 (2.5%)
Education	
Some high school or less	12 (30.0%)
High school graduate or GED	14 (35.0%)
Some college or technical school	13 (32.5%)
Unknown/not reported	1 (2.5%)
Employment	
Employed full time	2 (5.0%)
Employed part time	4 (10.0%)
Out of work for more than 1 year	8 (20.0%)
Out of work for less than 1 year	7 (17.5%)
Unable to work or disabled	19 (47.5%)
Income	
Less than $400 per month	17 (42.5%)
$400–$799 per month	15 (37.5%)
$800–$1199 per month	6 (15.0%)
$1200–$1799 per month	2 (5.0%)
Number of children	2.73 ± 2.21 (0–10)
MINI Psychotic Symptoms Score at Baseline	0.58 ± 1.11 (0–4)
Marijuana use ≥20 days in prior 30 days (n, % yes)	3 (7.5%)
Rost-Burnam Screener for Drug Abuse (n, % yes)	37 (92.5%)
Depressive Symptoms (PHQ-9)	7.38 ± 6.36 (0–23)
Perceived Stress (PSS-4)	6.35 ± 3.05 (1–13)
Anxiety (MINI)	2.13 ± 2.95 (0–9)
FTND Minutes to 1st Cigarette	
0–5 min	13 (32.5%)
6–15 min	8 (20.0%)
16–30 min	9 (22.5%)
31–60 min	6 (15.0%)
61+ minutes	4 (10.0%)
Alcohol-Use Severity (AUDIT-10 in Eligibility Survey)	14.93 ± 4.87 (7–24)

^a^*n* = 4 participants smoked < 5 CPD in the 7 days prior to the eligibility survey, but had missing data for their avg. CPD. For these participants, 2.5 CPD was assumed

### The stress of homelessness impacting smoking

Participants described the stress from being homeless as being an important factor in their smoking. For a number of participants, smoking had become a way of dealing with the stresses they were facing.

*This is kind of a stressful situation. People are homeless, being at the bottom of their luck, and—boom—and everything. So this is stress. What do you do? You drink, and you smoke, and that’s all that you can do, walking around here all day. Do you understand? (Participant 01,Male, African-American, intensive smoking intervention arm)*

Participants also described smoking as a way to pass the time, particularly if they were at or around the shelter for extended periods of time. Some found that they particularly wanted to smoke to cope with feelings such as anger and frustration that arose in dealing with their situation.

*Then it’s like life presents, and you need a cigarette! I might get mad or frustrated or whatever and I need a cigarette. Those are really my vulnerable times when I’m angry or frustrated, like I said, stressed out. (Participant 02, Male, African-American, standard care arm)*

### Challenges of the street and shelter environment and influence on smoking

Many participants described how cigarette smoke is common throughout the shelter and the areas near the shelter. They also described an active drinking community around the shelter. The ubiquitous nature of cigarettes and alcohol use in the homeless shelters creates an important opportunity for social interaction.

*It’s a hard thing to manage when you’re homeless. I think it’s harder when you’re homeless than anything, because it’s so vibrant and so concentrated around drinking, and smoking, and cigarettes, and stuff like that. (Participant 03, Male, More than 1 race, intensive smoking intervention arm)*

Participants described the physical environment, in particular the smell of cigarette smoke, as a factor that made it difficult to quit. While observing other people drinking might increase the temptation to drink, the smell of cigarette smoke and the sight of people smoking, was much more common, and difficult to avoid.

*Drinking ain’t hard to stay away from, it’s the smoking because smoking, you see that everywhere. (Participant 04, Male, African-American, intensive smoking intervention arm)*

Many participants described feeling pressured to partake in smoking from the culture of smoking found in the shelter communities. They stated that this pressure from the community and interpersonal relationships made it increasingly difficult for them to quit smoking while living in homeless shelters.

*If I am around a bunch of people, and they are smoking. That will make me want to smoke. (Participant 05, Male, African-American, intensive smoking and alcohol intervention arm)*

Several participants reported that they did not smoke before entering the shelters, but within a short period they picked up the behavior. Others recounted that they had stopped smoking but started smoking again after being in the shelter. Participants also described an increase in their smoking during the period of homelessness.

### The relationship between smoking and housing

The social culture of smoking led some participants to describe being so focused on smoking that it negatively impacted the use of their time, taking focus away from finding housing. For those who did have jobs, some described that their non-work time was focused on relaxing which often meant smoking. For participants that reported some degree of success in quitting, they frequently described a beneficial impact on the subsequent availability of more time to focus on obtaining housing.

*(Quitting) helped me focus more on my housing and stuff and not looking to go get a drink or smoke some cigarettes or something. (Participant 06, Male, African-American, standard care arm)*

Quitting was also described as financially rewarding as some participants put the money they would have used for smoking, into housing.

*You need to find a house. Not saying I’m better than anybody, but that was my goal, and I was thinking like ‘Man, I’m spending eight dollars on cigarettes! I could be putting that in a jar, helping me get a house!’ (Participant 07, Female, African-American, intensive smoking intervention arm)*

Many of the participants described that once they were away from the social and physical environment of the homeless shelters it was easier to stay quit because there is no longer the community and interpersonal pressure to smoke and drink. Housing was very important to most of the participants who were interviewed, and some expressed desire for the smoking cessation study staff to also help with finding housing. Some participants felt that if they were given housing with this research project, that they would have been more successful in their process of quitting.

### Motivation to quit smoking

Despite the challenges of trying to quit smoking while dealing with the stress of homelessness and the culture of smoking around shelters, the participants described feeling motivated to try to quit smoking. Participants described the need to have personal will, motivation and determination to successfully quit smoking. Many participants described wanting to quit and having a will and drive to quit.

*My goal is to quit within a month or two months. I talked to a couple of people. ‘It ain’t going to happen.’ I said, ‘well if you set your mind to certain things you can do this.’ (Participant 08, Male, African-American, intensive smoking and alcohol intervention arm)*

Some participants were particularly motivated by the health benefits of stopping smoking and by a desire to live longer and free of smoking-related disease. Some were experiencing illnesses that would be positively impacted by quitting.

*Well, first of all, I have a heart condition going down on this, so I need to quit. So that’s my main motivation because it causes getting chest pains. (Participant 01,Male, African-American, intensive smoking intervention arm)*

Others described wanting to quit smoking because they had become grandparents, they wanted to live longer to enjoy their grandchildren, and they didn’t want to smoke or smell like smoke around these children and babies.

*My grandkids was getting like, ‘Man, you smell like cigarette smoke.’ I had two new grandkids, so I thought it was time for me to stop.* (*(Participant 37, Male, African-American, intensive smoking intervention arm)*

Many of the participants described being very proud of meeting their personal goal of cutting down on cigarettes, even though the PTQ2 program had encouraged total cessation. Many described being down to one or two cigarettes per day, and some had made large reductions they felt proud of, even if they were still smoking half a pack of cigarettes a day.

*You know what? I can say, and I’m very glad to tell you guys when I first started this program, I was smoking two packs or I’m going to say a pack and a half a day with coffee. Now it’s like a half a pack. (Participant 09, Female, African-American, standard care arm)*

## Discussion

Our data highlights how reciprocal interactions among cognitions, behaviors, and the environment influence smoking cessation for people experiencing homelessness. The literature has already documented that shelters can be a challenge for smoking cessation as they are a pro-smoking normative environment [10], where social interactions with peers that are oriented around smoking, can be a barrier to cessation [9, 10]. The participants in this study described feeling pressure to smoke and drink in and around shelters, and that this pressure had led to some to start smoking or resume smoking, along with making it very challenging to quit. The pressure is extensive and it raises a challenge for environments such as shelters to address smoking as a problem in shelter environments, and to also address the need for alternative ways for residents to interact socially and collectively, in spaces and ways that do not promote pro-smoking norms.

This group of participants also expressed the value of smoking cessation, along with alcohol cessation, for positively impacting the time and focus they felt they had for finding housing. This suggests that addressing smoking among people experiencing homelessness is not just important in relation to reducing mortality and morbidity [4], but also may be seen as priority for supporting the search for housing, including through saving money to direct towards housing goals. This challenges the idea that homelessness would be further exacerbated by attempts at quitting [12], and asserts that smoking cessation or reduction could be seen as an important priority to integrate alongside strategies that are focused on finding housing, in order to more fully address the needs of people experiencing homelessness.

The motivation to quit smoking often is underestimated by professionals providing care for homeless smokers [14–16]. Notably, our study participants expressed a great deal of motivation and drive to address their smoking, yet they didn’t express as much enthusiasm for the idea of complete cessation. Many participants felt they had proudly achieved the best goal they could in the context of their current circumstances. This prompts the need to reflect on the goal of cessation programs and studies when working with people experiencing homelessness. Is cessation a goal that people experiencing homelessness share, or can it only be fully achieved in the context of also addressing the normative culture of shelters and the need for stable housing? Studies may be needed to follow smokers longer-term to see how reduction achieved while experiencing homelessness may impact future smoking behavior.

### Limitations

This study has several limitations. Participants are smokers experiencing homelessness who volunteered to participate in smoking cessation study, and therefore may have some self-selecting views in favor of smoking cessation. Results may not generalize to those who lack some desire to stop smoking. Additionally this sample reflects a group of individuals located in the Upper Midwest of the United States, and may not be representative of the view and concerns in other communities.

## Conclusions

Addressing smoking cessation among people experiencing homelessness is both an important public health opportunity, and a challenge. There is a need to consider participation in smoking cessation in the context of the social and environmental factors impacting smokers who are experiencing homelessness. In particular there is a need to address the impact of the shelter environment and the collective value placed on smoking in social interactions. Despite these challenges there are clearly high levels of motivation and interest in addressing smoking. Future research is needed to better understand the value of smoking reduction and its impact on individuals as they gain stability in housing.

## Abbreviations

CBT: Cognitive behavioral therapy
MI: Motivational interviewing
PTQ2: Power to Quit 2
